# Niclosamide loaded biodegradable chitosan nanocargoes: an *in vitro* study for potential application in cancer therapy

**DOI:** 10.1098/rsos.170611

**Published:** 2017-11-08

**Authors:** Saba Naqvi, Shanid Mohiyuddin, P. Gopinath

**Affiliations:** 1 Nanobiotechnology Laboratory, Centre of Nanotechnology, Indian Institute of Technology Roorkee, Roorkee, Uttarakhand 247667, India; 2 Department of Biotechnology, Indian Institute of Technology Roorkee, Roorkee, Uttarakhand 247667, India

**Keywords:** chitosan, niclosamide, cell cycle, semi-quantitative reverse transcription polymerase chain reaction, apoptosis

## Abstract

Chitosan nanoparticles can advance the pharmacological and therapeutic properties of chemotherapeutic agents by controlling release rates and targeted delivery process, which eliminates the limitations of conventional anti-cancer therapies and they are also safe as well as cost-effective. The aim of present study is to explore the anti-tumour effect of niclosamide in lung and breast cancer cell lines using biocompatible and biodegradable carrier where nanoparticles loaded with hydrophobic drug (niclosamide) were synthesized, characterized and applied as a stable anti-cancer agent. Niclosamide loaded chitosan nanoparticles (Nic-Chi Np's) of size approximately 100–120 nm in diameter containing hydrophobic anti-cancer drug, i.e. niclosamide, were prepared. Physico-chemical characterization confirms that the prepared nanoparticles are spherical, monodispersed and stable in aqueous systems. The therapeutic efficacy of Nic-Chi Np's was evaluated against breast cancer cell line (MCF-7) and human lung cancer cell line (A549). MTT assay reveals the cell viability of the prepared Nic-Chi Np's against A549 and MCF-7 cells and obtained an IC_50_ value of 8.75 µM and 7.5 µM, respectively. Acridine orange/ethidium bromide dual staining results verified the loss of the majority of the cells by apoptosis. Flow cytometer analysis quantified the generation of intracellular reactive oxygen species (ROS) and signified that exposure to a higher concentration (2 × IC_50_) of Nic-Chi Np's resulted in elevated ROS generation. Notably, Nic-Chi Np treatment showed more apoptosis and cell death in MCF-7 as compared to A549. Further, the remarkable induction of apoptosis by Nic-Chi Np's was confirmed by semi-quantitative reverse transcription polymerase chain reaction, scanning electron microscopy and cell-cycle analysis. Thus, Nic-Chi Np's may have a great potential even at low concentration for anti-cancer therapy and may replace or substitute more toxic anti-mitotic drugs in the near future.

## 1. Introduction

Nanotechnology has encouraged advances across several industries including clean energy to therapeutic drug delivery. The unique mechanical, physical and chemical properties of nanomaterials underscore the enormous potential of nanotechnology to play a vital role in healthcare. Various types of nanoparticulate drug formulations like polymeric micelles [1,2] and core shell type nanofibres [3,4] have been studied for hydrophobic and hydrophilic drugs. Biodegradable polymers have always a cutting edge over other non-biodegradable polymers due to their ability to overcome toxicity issues and least immunogenic response. Chitosan (polysaccharide) is considered as the most promising natural carrier due to its excellent biocompatibility, biodegradability and thus proved to be excellent in gene delivery [5] and anti-cancer therapy [6], and as a haemostatic, fungistatic, spermicidal, anticholesteremic, central nervous system depressive and immunoadjuvant agent [7]. Chitosan is derived via removal of an acetate moiety from chitin through deacetylation reaction in concentrated alkali. Chitosan is a copolymer of glucosamine and *N*-acetyl glucosamine consisting of β-(1,4)-2–amino-2-deoxy-d-glucopyranose (glucosamine units) and β-(1,4)-2-acetamido-2-deoxy-d-glucopyranose (acetyl glucosamine units). Physiochemical properties of chitosan are mainly decided by its degree of deacetylation and molecular weight. It is soluble in water only in acidic conditions, whereas its glycosidic bond which is hemiacetal in nature is hydrolysed and acquires positive charge after amino group protonation, thus leading to a decrease in molecular weight as well as viscosity. The presence of a reactive amino group in the pyranose monomeric unit of chitosan makes it an excellent candidate for the role of a carrier of biologically active compounds [8].

Niclosamide is a current chemotherapeutic agent that was discovered as a result of pre-clinical screening of compounds for cytotoxic potency *in vitro* against murine and/or human cancer cells [9,10] or *in vivo* model [11]. Niclosamide is a US Food and Drug Administration approved drug and has been exploited for its anti-helminthic properties in medicine for human welfare [12,13]. Anti-helminthics are medicines used in the treatment of worm infections. Parasitic worms of the flatworm phylum commonly referred to as cestodes are basically dependent on aerobic metabolism; from a literature review it is revealed that niclosamide inhibits oxidative phosphorylation in the mitochondria of cestodes. Recent studies in the past few years have shown the potential of niclosamide as a promising therapeutic anti-cancer drug. Studies suggested that anti-proliferative and apoptosis inducing property of niclosamide is due to LRP6 degradation by inhibiting Wnt/b-catenin signalling [14]. In addition, studies also indicated its radiosensitizer property [15]. Various pathways have been suggested for its anti-tumour activity where it blocks multiple signalling pathways (Wnt/β-catenin [16,17], mTORC1, Stat3 [18], NF-κB [11], Notch) and induces cell-cycle arrest via targeting mitochondrial enzymes with growth inhibition leading to programmed cell death.

Breast and lung cancers still remain some of the most devastating health threats which lead to huge mortality among people worldwide. Among most cancer types like prostate, colon and rectum, leukaemia, liver and pancreas, the current cancer incidence rate is about 20% higher in men than women. Chemotherapy is one of the conventional methods to treat cancer but it has many adverse side effects. According to cancer statistics report, it is estimated that almost 1.7 million new cases of cancer will be diagnosed in 2017. Lung cancer is the second most common cancer after prostate (19%) cancer in men. On the other hand, among females, breast (30%), lung (12%) and colorectal (8%) cancers are the most commonly diagnosed (a study from Cancer Statistics: 2017 by American Cancer Society). Therefore, there is a current need to develop new efficacious anti-cancer drugs having low toxicity drug cargoes. Engineered biodegradable nanomaterials have recently been emerging as attractive pharmacological vehicles for drug delivery and cancer therapy. Chitosan is a biodegradable and biocompatible polymer and niclosamide is nowadays a well-known anti-cancer drug; thus by combining these two into a single platform by exploiting nanotechnology, we synthesized new cost-effective, low adverse toxicity and high therapeutic index nanoformulations of hydrophobic drug.

The aim of this study was to develop agents that can modulate or inhibit molecular targets identified as being vital for tumour growth, and niclosamide loaded chitosan nanoparticles (Nic-Chi Np's) proved to be an effective anti-tumour agent owing to their well-established biodegradable and biocompatible nature.

## 2. Experimental

### 2.1. Materials

Chitosan (molecular weight of 190 000–375 000), niclosamide, glutaraldehyde, sodium sulfate, and sodium metabisulfite were purchased from Sigma-Aldrich (USA). Glacial acetic acid was purchased from SD-fine chemicals limited (SDFCL), India. All the solutions were prepared in ultra-pure Milli-Q water. All chemicals and reagents used for this study are of analytical grade and purchased from Sigma-Aldrich or Merck (Darmstadt, Germany).

### 2.2. Synthesis of niclosamide loaded chitosan nanoparticles

For the synthesis of Nic (niclosamide) loaded Chi (chitosan) nanoformulations, we followed desolvation method [19]. In brief, in a 10 ml chitosan solution (Sigma-Aldrich, USA) dissolved in dilute acetic acid (35 mM), Polysorbate 80 (Sigma-Aldrich, USA) (non-ionic surfactant: 230 µl) was added with constant stirring for 1 h at 440 r.p.m. After that, 350 µl of anti-cancer drug (Nic; stock: 1 mg ml^−1^ in ethanol) (Sigma-Aldrich, USA) was added dropwise into 10 ml of 0.15% chitosan solution and left stirring for another 1.5 h. Following that, 200 µl of 20% sodium sulfate (Sigma-Aldrich, USA) solution was added dropwise with further stirring for another 1 h. In order to cross-link the formed nanoparticles 80 µl glutaraldehyde (50% solution) (Sigma-Aldrich, USA) was added to the solution and stirring continued for another 1 h. The concentration of glutaraldehyde and sodium sulfate has a great impact on the size of formed nanoparticles. Lastly, sodium metabisulfite (10%) (Sigma-Aldrich, USA) was added to the nanoparticulate solution and stirred for another 2–3 h. All reactions were carried out in cold conditions at 4°C followed by dialysis against water with two changes and against normal saline with two changes for the next 48 h. The solution was lyophilized and preserved at 4°C for further characterization and therapeutic studies.

### 2.3. Encapsulation efficiency of niclosamide loaded chitosan nanoformulations

After the synthesis of Nic-Chi Np's, they were lyophilized at −80°C and 0.085 mbar pressure by a freeze dryer to get a lyophilized nanoparticle powder. A known amount of lyophilized nanoparticles were then redispersed in ethanol and ultrasonicated for 30 min. After centrifugation, the resultant released drug remained in the supernatant and was analysed by a UV-4 visible spectrophotometer (Lasany double-beam L1 2800) and the entrapped drug was calculated by using calibration curve of niclosamide in ethanol. The EE was calculated according to the formula

$$E(\%)=\left(\frac{{\left[\text{Drug}\right]}_{\mathrm{total}}-{\left[\text{Drug}\right]}_{\mathrm{free}}}{{\left[\text{Drug}\right]}_{\mathrm{total}}}\right)\times 100.$$

### 2.4. Physical characterization of nanoparticles

#### 2.4.1. Transmission electron microscopic pictures

Transmission electron microscopy (TEM) studies were carried out to determine the size, shape and morphology of synthesized nanoparticles. Briefly, lyophilized Nic-Chi Np's were dispersed in distilled water, and take 5 µl of the aqueous dispersion of Nic-Chi Np's followed by addition of 5 µl of 1% phosphotungstic acid. Then, nanoparticle solution mixture was put on a formvar coated copper grid (1% solution of formvar was prepared in spectroscopic grade chloroform) and air-dried in a vacuum desiccator [20]. The dried grid was examined under an FEI TECHNAI G2 model electron microscope unit operating at 200 keV at IIC, Indian Institute of Technology, India.

#### 2.4.2. Particle size and surface charge determinations

A Malvern Zetasizer measured the size of Nic-Chi Np's at 25°C using dynamic light scattering (DLS) and zeta potential. One milligram of lyophilized Nic-Chi Np's was dispersed in 2 ml distilled water by sonication. The size and zeta potential were measured at 1.34 refractive index and 0.2 absorbance using a cuvette. The size distribution obtained from photon correlation spectroscopy (PCS) is based on the intensity of scattered light [20].

#### 2.4.3. Morphology determination

Morphology and size of the nanoparticles were investigated using field-emission scanning electron microscopy (FE-SEM; Carl Zeiss ULTRA PLUS) and atomic force microscopy (AFM; NTEGRA PNL) operating in semi-contact mode. An aliquot of Nic-Chi Np suspension was layered on a glass coverslip and dehydrated for 15–20 min in an oven at 25–30°C. Imaging was performed with FE-SEM and AFM at different spots on the dried nanoparticles. The images of AFM were further processed using NOVA software.

#### 2.4.4. Crystalline characteristics of the particles by X-ray diffraction studies

Bruker AXS D8 Advance X-ray diffractometer using Ni filtered Cu-Ka radiation was used for X-ray diffraction (XRD) studies with a scan speed of 0.05° min^−1^ over the range of 2*θ* = 5–90°. Two mg of lyophilized nanoparticles was used for XRD analysis. The obtained XRD patterns were analysed by using PANalytical X’ Pert High score software.

#### 2.4.5. Fourier transform infrared spectroscopic analysis

Fourier transform infrared (FTIR) analysis was performed for drug alone (niclosamide), Nic-Chi Np's, and chitosan powder, in order to confirm the presence of covalent interaction between various components of the polymer matrix and the drug loaded within them. It is based on the vibrations of atoms in a molecule. The presence of such covalent interaction can possibly introduce unfavourable alterations in drugs and might compromise their therapeutic efficacy. Apart from this, FTIR analysis was also carried out to verify completion of glutaraldehyde-mediated cross-linking reaction. The FTIR spectra were recorded by a Thermo Nicolet spectrometer using the KBr pellet technique in the 4000–400 cm^−1^ range with 32 scans.

#### 2.4.6. Thermogravimetric analysis

Thermal stability of Nic-Chi Np's was investigated by thermogravimetric analysis (TGA). In brief, about 10 mg of chitosan powder, niclosamide drug and Nic-Chi Np's were heated from 5°C to 600°C at a constant rate of 10°C min^−1^ in an EXSTAR TG/DTA 6300. A constant nitrogen atmosphere was maintained throughout the TGA of all samples.

### 2.5. *In vitro* drug release study of niclosamide loaded chitosan nanoparticles

The drug releases from Nic-Chi Np's were studied *in vitro* by a Slide-A-Lyzer™ MINI dialysis device (10 kDa MWCO) at pH 5.5 in sodium acetate buffer and pH 7.4 in phosphate-buffered saline (PBS). Briefly, 4 ml of dispersed Nic-Chi Np's (440 µg) was added to the inner tube and the outer compartment was filled with 45 ml of similar release media (pH 5.5 and pH 7.4). The dialysis devices were gently agitated at 120 r.p.m at 37°C in incubator shaker. At predetermined time intervals of 3, 6, 9, 12, 24, 36 and 48 h, 1 ml of buffer solution containing Nic-Chi Np's was withdrawn from the outer compartment and replaced with 1 ml of fresh media of the same pH to maintain sink conditions. The concentration of released niclosamide in the medium was quantitated by measuring the absorbance at 346 nm using a UV–visible spectrophotometer. The *in vitro* drug release assay was performed thrice. The drug percentage release was calculated as follows:

$$\%\text{ Release }=\left[\frac{{\text{Conc. of Drug}}_{\mathrm{aliquot}}}{\text{Initial Drug Conc.}}\right]\times 100.$$

### 2.6. Cell culture studies: *in vitro* anti-cancer studies

A549 and MCF-7 (human lung and breast adenocarcinoma cell lines, NCCS Pune) were maintained in Dulbecco's modified Eagle's medium (DMEM) supplemented with 10% fetal bovine serum (Gibco Life Technologies, UK) supplemented with 1% penicillin–streptomycin (Sigma-Aldrich, USA) and maintained at 37°C in a humidified atmosphere with 5% CO_2_. Cells were subcultured at every 48 h by using 0.25% trypsin–EDTA and harvested until subconfluency reached 60–70%.

#### 2.6.1. Cell cytotoxicity/viability test

The cytotoxic effect of Nic-Chi Np's was evaluated by well-established cell viability 3-(4,5-dimethylthiazol-2-yl)-2,5-diphenyltetrazolium bromide (MTT) assay. Briefly, A549 and MCF-7 cells were plated at a density of 10^4^ cells per well into 96-well plates in complete culture medium until sub-confluent (Corning, Costar, NY, USA). The cells were then incubated with different low to high concentrations of the void chitosan nanoparticles (VCNp) and Nic-Chi Np's for 24 h. After incubation, cells were washed with sterile PBS, media removed and 90 µl of fresh DMEM media was added per well to the cells. MTT (Sigma-Aldrich, USA) dye (10 µl reagent; 5 mg ml^−1^ stock) was then added per well including controls, and the plate returned to the cell culture incubator for 3–4 h. After incubation, the cells were examined under an inverted microscope for the appearance of an intracellular punctate purple precipitate. After the formation of the purple precipitate, media was discarded from the wells and 100 µl of dimethyl sulfoxide was added to solubilize the formazan crystals formed, including controls. The plate was left for mixing in the dark for 20–30 min, with gentle swirling, but not shaking. The absorbance was measured in each well, including blank using a multimode reader (Cytation3, Biotek) at 570 nm and the background control at 690 nm. All the measurements were performed in triplicate.

Cell viability (%) was calculated as

$$\text{Cell viability}=\frac{{\left(\text{A570-A690}\right)}_{\mathrm{treated}}}{{\left(\text{A570-A690}\right)}_{\mathrm{control}}}\times 100,$$

where A570 and A690 are the absorbance values obtained at 570 nm and 690 nm, respectively.

### 2.7. Cell morphology study by fluorescence microscopy

#### 2.7.1. Acridine orange/ethidium bromide dual staining

Dual acridine orange/ethidium bromide (AO/EB) fluorescent staining of Nic-Chi Np's against A549 and MCF-7 cells was visualized under a fluorescent microscope [21]. Briefly, 2 × 10^5^ cells were seeded in each well of 6-well culture plates and plates were incubated overnight. Cells were then left untreated and treated with IC_50_ and 2 × IC_50_ Nic-Chi Np's for around 24 h. Following treatment, cells were washed with ice cold PBS and each well was incubated with dual AO/EB fluorescent staining solution (10 µg ml^−1^ working concentration) (Sigma-Aldrich, USA) at 37°C for 8–10 min. Subsequently, one PBS wash was done to remove excessive dye (to avoid background fluorescence of free dye). Images of stained cells were counted within 15–20 min and examined with an EVOS cell imaging system (Life Technologies, USA). Dual AO/EB staining protocol was repeated three times at least.

#### 2.7.2. Time-dependent morphological examination using fluorescent dyes Hoechst 33342 and rhodamine B

Cytoskeletal changes and nuclear chromatin compaction with respect to time were monitored in MCF-7 and A549 cell lines with the addition of fluorescent dyes Hoechst 33342 (Life Technologies, USA) and rhodamine B (Rho B). MCF-7 and A549 cells were plated at a density of 2 × 10^5^ cells well^−1^ in 6-well plate followed by treatment with Nic-Chi Np's at different time points of 6, 12 and 24 h. After different incubation time cells were washed with PBS in order to remove dead floating cells followed by addition of 2 µl Hoechst dye (stock conc: 10 mg ml^−1^) and 3 µl Rho B (stock conc: 1 mg ml^−1^) for 8–10 min at 37°C. An overlay of images was examined by EVOS cell imaging system under DAPI filter and red filters according to their respective excitation and emission wavelengths (Rho B at an excitation of 540 nm and emission of 625 nm and Hoechst 33342 dye at an excitation of 352 nm and emission of 460 nm). Time-dependent morphological examination using fluorescent dye method was repeated thrice at least.

### 2.8. Cell morphology analysis by field-emission scanning electron microscopy

FE-SEM analysis was carried out for examining the cell morphology of cancer cells after treatment with Nic-Chi Np's. MCF-7 cells (2 × 10^5^ cells) and A549 cells (2 × 10^5^ cells) were seeded on glass coverslips in 35 mm plates and treated with Nic-Chi Np's at their respective 2 × IC_50_ for 12–16 h. PBS wash was given after incubation time and cells fixed with 2% glutaraldehyde solution for 5–10 min followed by ethanol gradient fixation (20%, 40%, 60% and 80%). Cells were analysed under FE-SEM (Ultra plus, Carl Zeiss) operating at 5 kV. Elemental analysis of drug-loaded chitosan nanoparticles was also done by FE-SEM.

### 2.9. Intracellular reactive oxygen species detection by flow cytometry

Here, we verified the redox state of MCF-7 and A549 cells by flow cytometry technique in order to detect reactive oxygen species (ROS) production with fluorescent probes. We performed the 2′,7′-dichlorodihydrofluorescein diacetate (DCFH-DA) method for measuring oxidative stress in MCF-7 and A549 cells due to its advantages over other methods like it being easy to perform and analyse, cost-effective and extremely sensitive to changes in the redox state of a cell over time. MCF-7 and A549 cells were seeded in a 6-well plate until sub-confluent, and were treated with defined IC_50_ concentration of Nic-Chi Np's for 12 h. Intracellular ROS was measured using a peroxide-sensitive fluorescent probe, carboxy-2′, 7′-dichlorofluorescein diacetate (H_2_DCFDDA; Sigma-Aldrich). Cells were then treated with 20 µM DCFH-DA dye dispersed in 1 ml fresh DMEM and incubated for 10 min at 37°C. Following incubation, cells were extracted and resuspended in sterile PBS. Samples were analysed for 2,7-dichlorofluorescein (DCF) fluorescence at excitation (495 nm) and emission maxima at 529 nm after incubation using a flow cytometer (Amnis Flowsight). The data were analysed by Amnis Ideas software for a total of 10 000 events per sample. Flow cytometry measures green fluorescence in ROS generated per cell.

### 2.10. Gene expression studies by semi-quantitative RT-PCR analysis

Semi-quantitative reverse transcription polymerase chain reaction (RT-PCR) was performed for apoptotic gene expression studies. The treated MCF-7 and A549 cells were harvested from the Nic-Chi Np's up to 24 h incubation. Further, the total RNA from the cells was isolated by using the TRI reagent (Sigma-Aldrich, USA) RNA isolation method. The isolated total RNA was qualitatively determined by 1.2% agarose gel electrophoresis under UV illumination of ethidium bromide staining. This RNA was used as a template for cDNA synthesis; it was achieved by reverse transcription reaction using Super Script II Reverse Transcriptase (Invitrogen, California, USA). The qualitative gene expression levels of apoptotic genes are estimated by sampling the cDNA product with gene specific primer for RT-PCR (Applied Biosystems) amplification. The amplified products of RT-PCR were run along with the 1 kb ladder DNA (ProxyO 100 bp DNA ladder, SRL Pvt. Ltd, India) loaded in 1.2% agarose gel and visualized under UV light by ethidium bromide staining. Fold alteration in gene expression was approximated by Image lab 5.2.1 software while considering untreated control samples as absolute. The pro-apoptotic set of genes used for gene expression studies included p^53^, Bax, Bad, Caspase-3, while the anti-apoptotic genes were Bcl-2 and Bcl-xL. β-Actin (housekeeping gene) was engaged as an internal control. The primer pairs of both pro- and anti-apoptotic genes were elucidated as shown in the electronic supplementary material, table S1.

### 2.11. Cell-cycle analysis

For cell-cycle analysis we did flow cytometry, which is a classical method to detect cell apoptosis, where cells were stained with propidium iodide (PI) [22]. MCF-7 cells were seeded at a density of 2 × 10^5^ cells per well into 6-well plate in DMEM culture medium. When cells were 70–80% confluent, they were treated with an IC_50_ concentration of Nic-Chi Np's for 24 h. Following treatment, cells were washed with ice cold sterile PBS, and harvested by trypsinization and centrifuged for 5 min at 500*g* at 4°C. DMEM (1 ml) was added to stop trypsinization and cell pellet was then fixed with 70% ethanol and then placed in the centrifuge tubes on ice for at least for 15 min. PI (Sigma-Aldrich, USA) staining solution (50 mg ml^−1^ PI, 1 mg ml^−1^ RNase A and 0.05% Triton X-100) was added into fixed cells and incubated for 50 min at 37°C in the dark followed immediately by flow cytometry analysis (Amnis Flowsight). A total of 10 000 events were analysed by flow cytometry.

### 2.12. Statistical analysis

The data were processed using Microsoft Excel, graphed with Prism 5 software (GraphPad, v. 6.1). Statistical analyses were performed using the Student's *t*-test for unpaired data or by two-way ANOVA whichever was applicable and *p* < 0.05 was considered statistically significant. Data are presented as mean ± standard error of the mean.

## 3. Results and discussion

### 3.1. Synthesis of niclosamide loaded chitosan nanoparticles and UV–visible spectroscopy

**Figure 1.**
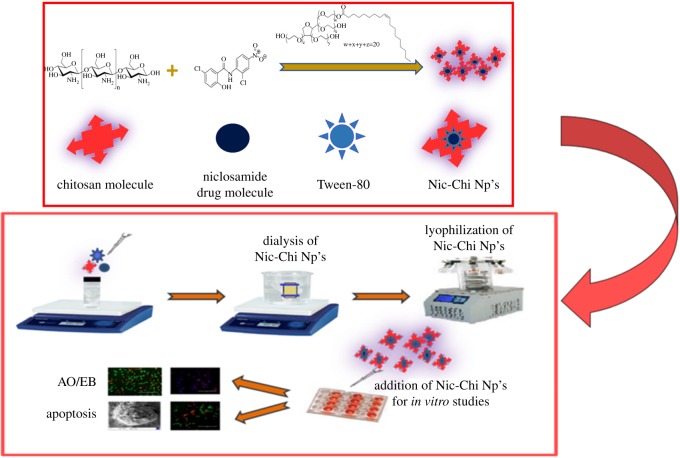
Schematic of synthesis of Nic-Chi Np's followed by effective anti-cancerous effect *in vitro* studies.

**Figure 2.**
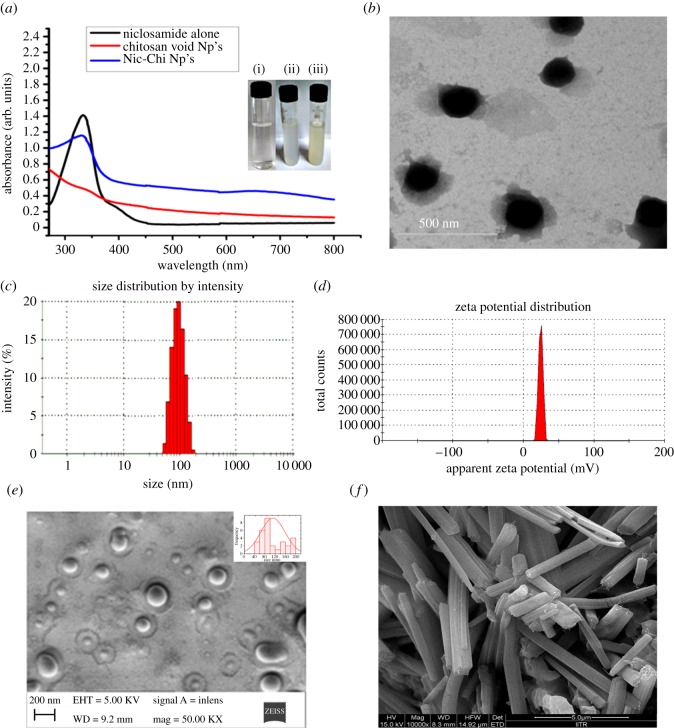
(*a*) UV–visible absorption spectra of niclosamide alone (in ethanol), chitosan void nanoparticles, i.e. without drug, and Nic-Chi Np's, i.e. chitosan nanoparticles loaded with drug niclosamide. Representative images of (i) chitosan clear solution before nanoparticle synthesis; (ii) chitosan void Np's; (iii) chitosan nanoparticles loaded with drug niclosamide. (*b*) TEM image; (*c*) dynamic light scattering measurement; (*d*) zeta potential on the niclosamide loaded chitosan nanoparticles (Nic-Chi Np's). FE-SEM images of (*e*) Nic-Chi Np's and (*f*) raw niclosamide powder, showing their typical morphology.

Hydrophobic anti-cancer drug, i.e. niclosamide, was encapsulated in chitosan cargo cross-linked with glutaraldehyde using mild synthetic conditions. Nic-Chi Np's were prepared by desolvation method using sodium sulfate as precipitating agent. The mixture of chitosan and P-80 was stirred and dropwise addition of sodium sulfate into the mixture results in desolvated chitosan into particulate form; further glutaraldehyde cross-linking of the nanoparticles and addition of sodium metabisulfite lead to stable particles. The presence of free amino groups, as well as hydroxyl groups of chitosan, enables different chemical modifications to produce a relatively more rigid and robust chitosan structure. Thus, a reactive amino group of chitosan undergoes a covalent cross-linking with the aldehyde group of glutaraldehyde. Tween-80 (P-80) is used as a stabilizer for the suspension. An addition of Polysorbate 80 was required to stabilize the nanoparticle solution; without Polysorbate 80 the growth of agglomerates occurred. Figure 1 shows the schematic presentation of synthesis of nanoparticles which were further used for *in vitro* cell culture studies. The entrapment efficiency of the drug is found to be greater than 90%. The Nic-Chi Np's were further analysed with UV–visible spectroscopy in the spectral range of 260–800 nm. We observed the characteristic peak of niclosamide drug alone when dissolved in ethanol at 346 nm (figure 2*a*). This peak is also reported in drug-loaded chitosan nanoparticles but it is absent in void nanoparticles, hence from absorption spectral peak, it is clear that drug is encapsulated inside the chitosan nanoparticles. On the other hand, free niclosamide is poorly soluble in aqueous media, having macroscopic undissolved flakes of the drug (as also reported in FE-SEM; figure 2*f*) visible in solution; by contrast, Nic-Chi Np's is a clear, dispersed formulation (figure 2*a*(i–iii)). However, there is a slight change in absorption spectra of niclosamide loaded chitosan nanoparticles seen in figure 2*a*, which may be due to chemical cross-linking between the –CHO group of glutaraldehyde and –NH_2_ group of chitosan molecules.

### 3.2. Physico-chemical characterization of the nanoparticles

The physico-chemical properties such as size, zeta potential and aggregation behaviour, crystalline or amorphous nature, thermal stability and chemical interactions of Nic-Chi Np's were studied by TEM, zeta sizer, XRD, TGA and FTIR, respectively.

#### 3.2.1. Particle size and size distribution

##### 3.2.1.1. TEM experiments

In our study, we synthesized Nic-Chi Np's by desolvation method using glutaraldehyde as cross-linking agent and the average size of Nic-Chi Np's was found to be, as shown in figure 2*b*, around 100–120 nm in diameter. The TEM picture of formed nanoparticles demonstrated spherical shape nanoparticles having a size below 120 nm in diameter (figure 2*b*).

##### 3.2.1.2. Dynamic light scattering experiments

The TEM result was further confirmed by PCS, i.e. DLS: measuring the size of dispersed Nic-Chi Np's provides the hydrodynamic radius on an average. Our results demonstrated that the size range of 120 nm with a maximum population of approximately 100 nm size particles were formed by desolvation method (figure 2*c*; electronic supplementary material, figure S1). The entire synthetic protocol was performed under cold conditions at 4°C with a controlled stirring rate. It was noted that higher temperature and longer duration of stirring, however, increase the particle size accordingly.

#### 3.2.2. Surface charge of nanoparticles

Zeta potential is an important physico-chemical parameter of nanocarriers which has a very vital effect in a drug delivery system. Zeta potential also decides the solubility and release rate of the nanoparticulate system as well as its cellular uptake in tumour microvasculature and blood circulation into the body. Our results showed that chitosan nanoparticles obtained by desolvation method were found to be (+)24 mV (figure 2*d*; electronic supplementary material, figure S2). The amino group of chitosan would be responsible for the positive ζ-potential. Chitosan when dissolved in water, only in acidic conditions, acquires positive charge after amino group protonation. Moreover, these amino groups are also freely assessable for interaction with niclosamide drug and glutaraldehyde for cross-linking. It was reported earlier by Berthold *et al*. [23] that molecular weight of chitosan had no effect on ζ-potential.

#### 3.2.3. Scanning electron microscopy and atomic force microscopy analyses

FE-SEM and AFM analyses were performed in order to confirm the morphology (shape and size) of synthesized nanoparticles. Figure 2*e* reports the spherical size of obtained nanoparticles with uniform size distribution. However niclosamide drug powder exhibits micrometre size rod shaped flakes in figure 2*f*; thus it is clear from SEM analysis that formed chitosan nanoparticles are spherical and drug is encapsulated inside the core of nanoparticles, which is further confirmed by elemental analysis of Nic-Chi Np's (electronic supplementary material, figure S3). It is again confirmed through energy-dispersive X-ray analysis of A549 and MCF-7 cells which exhibit chlorine inside cells when incubated with Nic-Chi Np's at different time periods (electronic supplementary material, figure S4). In AFM, the shape is round and mean diameter of average grain size of 120 nm (as is depicted by colour coding and three-dimensional image of nanoparticles) was observed (electronic supplementary material, figure S5). Thus, from TEM, DLS, FE-SEM, AFM and ζ-potential results, we conclude that obtained size is effective in anti-cancer property of Nic-Chi Np's due to their enhanced permeability and retention effect [24].

#### 3.2.4. Nanoparticle crystalline studies by X-ray diffraction analysis

**Figure 3.**
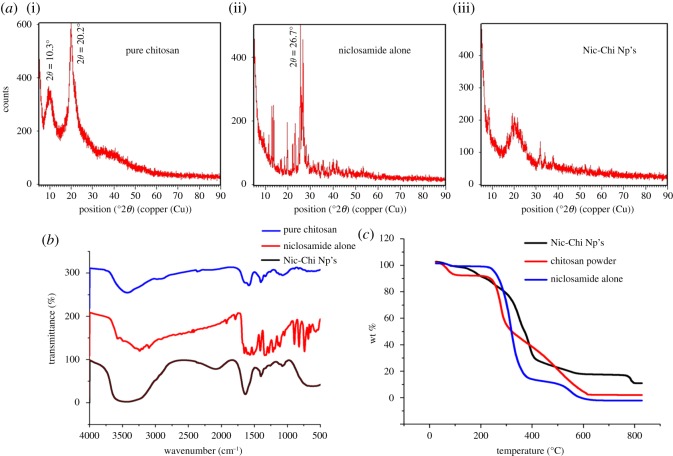
(*a*) XRD plot of (i) pure chitosan powder, (ii) niclosamide drug alone, (iii) loaded chitosan nanoparticles (Nic-Chi Np's). (*b*) FTIR analysis of pure chitosan powder, niclosamide drug alone and Nic-Chi Np's. (*c*) Thermogravimetric analysis (TGA) plot of pure chitosan powder, raw niclosamide powder and Nic-Chi Np's.

Crystal studies of chitosan powder, niclosamide drug alone and Nic-Chi Np's were done using XRD. The degree of deacetylation, molecular weight and polymer crystallinity are the parameters which describe the physico-chemical properties of chitosan polymer. Chitosan is semi-crystalline in nature and exhibits polymorphism depending on its physical state. Different crystal structure including anhydrous and hydrated was investigated by various scientists [25,26]; our results are corroborated with other findings. In figure 3*a*, we investigated the crystalline nature of chitosan powder and niclosamide drug alone but the drug-loaded chitosan nanoparticles were amorphous in nature. X-ray diffractogram of pure chitosan powder in figure 3*a*(i) exhibits characteristic reflections at 10.3° and 20.2° which is the typical fingerprint of semi-crystalline chitosan indexed as (020 and 110) intensity known for a crystalline and amorphous structure of chitosan, respectively. Niclosamide drug alone has sharp crystal peaks in figure 3*a*(ii), but they disappeared when it is encapsulated into the chitosan nanoparticles (figure 3*a*(iii)) which may be due to cross-linking mechanism among the reactive functional group (amine group) and drug molecules, along with the hydrogen bonding and electrostatic interaction between them. Moreover, the disappearance of sharp characteristic diffraction peaks of niclosamide [27] at 2*θ* = 25.6° and 26.7° in Nic-Chi Np's also confirms that drug was entrapped completely inside the core of chitosan nanoparticles.

#### 3.2.5. Fourier transform infrared analysis

FTIR is based on the vibration of atoms in molecules. Infrared electromagnetic radiation is passed through a sample having permanent and induced dipole moment and determines what fraction of the incident radiation is absorbed at that particular energy. The FTIR spectrum Nic-Chi Np's in the region between 100 and 2400 cm^−1^ showed the significant peaks for analysis. This is the region where the carbonyl, C─O–NHR, amine NH_2_ and ammonium, NH^3+^ band, OH and CH deformation are situated. Generally phenolic –OH groups absorb strongly at the stretching frequencies of 3700–3584 cm^−1^. In Nic-Chi Np's, chitosan showed a strong peak at 3400–3200 cm^−1^, corresponding to combined peak of the hydroxyl group (–OH), and intermolecular hydrogen bonding. A strong peak was observed in Nic-Chi Np's and void nanoparticles at 1636.66 cm^−1^, which is very small or negligible in pure chitosan but strong enough in nanoformulations which corresponds to C═N bond. This is attributed to the formation of Schiff base (imine), i.e. C═N, due to electrophilic carbon atoms of glutaraldehyde being targeted by nucleophilic attack of amine group of chitosan molecules. Stretching vibration of (C─O─H) is present at 1259.53 cm^−1^ in both loaded and VCNp backbone.

Absorption in the range from 1160 cm^−1^ to 1000 cm^−1^ has been attributed to vibrations of CO group [28]. Both asymmetric and symmetric stretching vibrations of (C─O─C) are present in chitosan nanoformulations in their backbone structure; the band located near 1124.12 cm^−1^ is related to asymmetric vibrations of CO in the oxygen bridge resulting from deacetylation of chitosan. The bands near 1079 cm^−1^ are attributed to _ν_CO of the ring COH, COC and CH_2_OH in loaded and void nanoparticles (figure 3*b*); electronic supplementary material, figure S6).

Characteristic infrared bands of organic halogen containing compounds like niclosamide which contain C─Cl bond are seen between 800 and 400 cm^−1^. Various peaks were reported in niclosamide (electronic supplementary material, figure S6) alone spectrum, i.e. 441.21, 470.65, 538.35, 576.61, 635.16, 684.93, 742.25 and we observed 414.72, 608.39 and 709.08 cm^−1^ peaks for Nic-Chi molecules. Moreover, the absence of absorption peaks corresponding to C─Cl group of niclosamide in IR spectra of VCNp also verifies the absence of drug molecule in VCNp. Furthermore, the absence of absorption peak corresponding to an electronegative NO_2_ group (1518.35 cm^−1^) of niclosamide in IR spectra and appearance of the strong NO_2_ stretch at 1403.76 cm^−1^ suggested electrostatic interaction during synthesis between the amine group of chitosan and NO_2_ group.

#### 3.2.6. Thermogravimetric analysis

Thermal degradation of Nic-Chi Np's, free niclosamide drug, and bare chitosan powder was studied by TGA in order to check thermal stability and percentage weight loss with respect to diverse temperature regimes (figure 3*c*). Our results showed different decomposition patterns of bare chitosan powder, Nic-Chi Np's and niclosamide alone. The initial loss of weight observed in chitosan powder ranging from 50°C to 100°C is ascribed to release of water molecules, but in niclosamide drug alone and Nic-Chi Np's, this initial weight loss did not occur, but at approximately 170°C a loss of weight was observed, because of the release of water molecules. From figure 3*c*, it is clearly depicted that Nic-Chi Np's start degrading gradually from 180 to 280°C due to loss of small molecules such as CO_2_, NH_3_, etc. However, there is abrupt decrease of weight loss observed from 200 to 420°C due to decomposition of chitosan, whereas in Nic-Chi Np's only 31% remains to 420°C and there is a gradual chitosan decomposition to 790°C, but the drug niclosamide was degraded drastically from 100 to 12% to 380°C and degraded completely at 600°C (i.e. only 0% weight is left). On the other hand, 18% Nic-Chi Np's were still remaining at 790°, whereas chitosan powder is also completely degraded (only 2–3% remains) at 800°C. Our results suggest that when drug is loaded inside nanoparticles and when nanoparticles were synthesized, their thermal degradation rate becomes slower and they are more stable as compared to bare drug and chitosan powder. This is ascribed to the higher rate of degradation of niclosamide alone and chitosan powder which may be due to free amino groups in chitosan but in niclosamide loaded chitosan, these free groups are used in cross-linking with the aldehyde group of glutaraldehyde, both by niclosamide and chitosan, and less or slower weight loss was reported.

### 3.3. *In vitro* drug release study of niclosamide loaded chitosan nanoparticles

**Figure 4.**
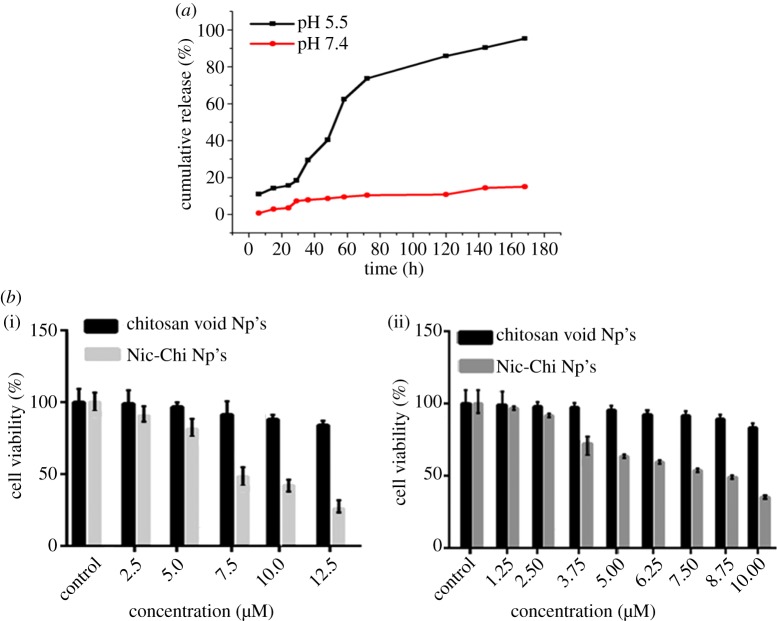
(*a*) Cumulative release profile of niclosamide from Nic-Chi Np's in PBS in acidic (pH = 5.5) and basic medium (pH = 7.4). (*b*) The effects of Nic-Chi Np's on cell proliferation and viability of (i) MCF-7 cells and (ii) A549 cells as determined by MTT assay. Concentration-dependent cytotoxic effects of nanoparticles were evaluated after 24 h incubation. Results are represented as mean ± standard error of the mean. *Significant difference from control (*p* < 0.05).

To simulate the tumour microenvironment and endosomal environment, *in vitro* Nic-Chi Np's drug release profile was monitored at physiological pH 7.4 and endosomal pH 5.5 (corresponding the pH of mature endosomes of tumour cells) with different time periods up to seven days. Figure 4*a* depicts Nic-Chi Np's as having pH responsive release profiles. We observed that niclosamide drug was more released under acidic conditions as compared to normal physiological pH conditions. Only approximately 15% drug was released up to 7 days and there was a gradual loss of drug observed at pH 7.4, whereas almost 90% of the loaded drug was released at pH 5.5. This may be due to protonation of an amino group of chitosan at lower pH value. Furthermore, our results support the therapeutic efficacy of Nic-Chi Np's showing enhanced anti-cancer effect as observed via *in vitro* experiments. We could say that less drug was released into the blood (pH 7.4) and majority of active drug was released from the core of Nic-Chi Np's after reaching the mildly acidic tumour microenvironment especially after the event of endocytosis.

### 3.4. *In vitro* cell culture studies

#### 3.4.1. Cell viability assay

Our group previously showed that bare niclosamide (in water) did not exhibit cytotoxicity in both A549 and MCF-7 cell lines [27], which may be owing to insolubility of niclosamide drug molecules in water as the solvent, hence resulting in rejection of drug molecules by cells. From Bharat *et al*. [27] study it was already clear that free niclosamide up to 14 µM concentration cannot exhibit significant toxicity. Thus, taking this into account we performed MTT assay of void and loaded chitosan nanoparticles only and not of niclosamide drug alone. A few studies demonstrated that chitosan itself as a carrier has an anti-cancer potential. In order to check that, we treated MCF-7 and A549 cells both with and without drug molecules. Our results demonstrated that there was a concentration-dependent cytotoxicity of Nic-Chi Np's in MCF-7 and A549 cells when treated with different concentration. In order to calculate IC_50_ for MCF-7 (figure 4*b*(i)), we tried at 2.5, 5, 7.5, 10, 12.5 (µM) concentration and found 7.5 µM to be IC_50_; however, in A549 (figure 4*b*(ii)), we tested 1.25, 2.5, 3.75, 5, 6.25, 7.5, 8.75 and 10 (µM) and found 8.75 µM as IC_50_. From the results, it is clear that when both breast cancer and lung cancer cells were kept at same incubation time, the IC_50_ of MCF-7 was reached at less concentration, i.e. 7.5 µM, as compared to A549, i.e. 8.75 µM. This may be due to MCF-7 being more susceptible to nanoformulations of niclosamide drug. MCF-7 is more effective in engulfing Nic-Chi Np's in less time as compared to lung cancer cell line; however, morphological examination of cells also confirms that when we increase the incubation time of nanoparticles from 8 to 16 h, MCF-7 starts to die, but A549 is not much affected (data not shown). An aqueous solution of nanoformulation of Nic-Chi can be taken by cells easily due to their positive charge over their surface, which further facilitates their cellular uptake by interaction with the plasma membrane. Chitosan nanoparticles can enhance anti-cancerous effect to a greater degree with a collaborative/additive anti-tumour effect of niclosamide. In our study, each concentration of void nanoparticles was evaluated along with drug-loaded nanoparticles which ensures that the anti-tumour effect of synthesized nanoparticles is due to anti-cancerous drug encapsulated in them and not due to chitosan carrier, although it was already demonstrated that free niclosamide up to 14 µM concentration cannot exhibit significant toxicity [27].

### 3.5. Cell morphology study by fluorescence microscopy

#### 3.5.1. Acridine orange/ethidium bromide dual staining

**Figure 5.**
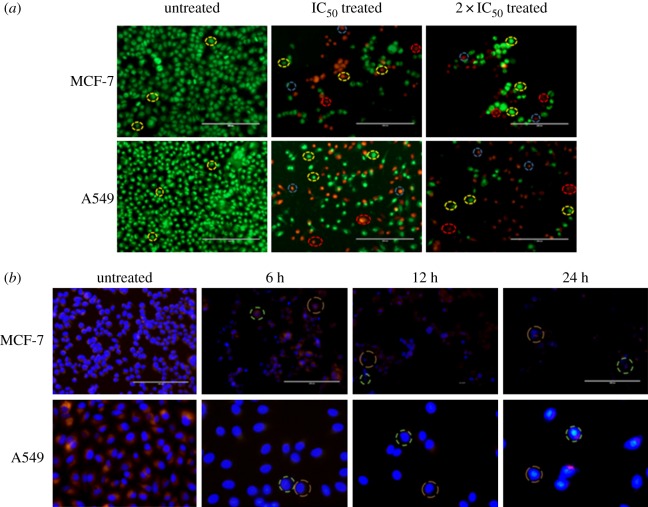
(*a*) Fluorescence microscopic images of AO/EB dual staining of untreated, IC_50_ and 2 × IC_50_ of Nic-Chi Np's treated MCF-7 and A549 cells. Yellow, blue and red dotted circles represent viable, early apoptotic and late apoptotic cells, respectively. Scale bar, 200 µm. (*b*) Time-dependent overlay images of untreated and Nic-Chi Np's (IC_50_) treated MCF-7 and A549 cells stained with Hoechst 33342 (blue) and co-stained with rhodamine B (red). Red doted circle indicates nuclear fragmentation, green shows cytoskeleton compaction. Scale bar, 200, 100 µm.

In order to differentiate/quantitate live, apoptotic cells from necrotic cells, one other approach we took in our study is AO/EB dual staining through which we are able to visualize nuclear changes and apoptotic body formation that are characteristic of the cascade of apoptosis. AO is a nucleic acid selective fluorescent cationic dye. It is cell permeable and will stain both live and dead cells. As compared to AO staining only, the AO/EB technique improves the detection of apoptosis and is able to distinguish between late apoptotic and dead cells. In control (i.e. untreated), live cells of MCF-7 and A549 appeared uniformly green (figure 5*a*); however, when treated with Nic-Chi Np's at IC_50_ (7.5 µM) and 2 × IC_50_ in MCF-7 and IC_50_ (8.75 µM) and 2 × IC_50_ in A549 cells, early apoptotic cells appeared stained green and contain bright green dots in the nuclei as a consequence of chromatin condensation and nuclear fragmentation; while 2 × IC_50_ treated cells displayed more late apoptotic cells and few necrotic cells. Owing to the presence of EB, only those cells can be stained that have lost membrane integrity. EB can only enter the membrane compromised nucleus to emit orange-red fluorescence. Thus upon incubation with Nic-Chi Np's at their estimated IC_50_ and double IC_50_ we demonstrated and confirm that normal cells and apoptotic cells can be easily distinguished by using this AO/EB staining and signifies apoptosis induction by Nic-Chi Np's in both in MCF-7 as well as in A549.

#### 3.5.2. Time-dependent morphological examination using fluorescent dyes Hoechst 33342 and rhodamine B

Subcellular localization of nucleic acids and nucleus was done by Hoechst to investigate the pycnotic nuclei in those cells which undergo apoptosis after incubation with Nic-Chi Np's at three different time points (6, 12, 24 h) (figure 5*b*).

Hoechst 33342 is a fluorescent DNA intercalating dye, stains apoptotic cells/nuclei and emits blue fluorescence when bound to dsDNA. It is clear from the morphological examination that, as the incubation time increases, the apoptotic nuclei appear small, fragmented and highly textured. In figure 5*b*, it is clearly depicted that in control, cells were healthy but as the incubation time of Nic-Chi Np's increases in both MCF-7 and A549, i.e. from 6 to 12 to 24 h, their nuclei start exhibiting chromatin condensation in the form of dark spots and a significant reduction in cytoplasmic volume was noted.

Rho B stained cells were examined under the green filter, which stains mitochondria and cytoplasmic vesicles, whereas Hoechst 33342 stains DNA and binds to the AT rich regions of dsDNA strands in the nucleus and enhance fluorescence approximately twofold greater than GC-rich strands observed under DAPI filter. We noticed that only a few Rho B stained cells were live after 24 h, and hence the appearance of several pycnotic nuclei suggested cell death induced in MCF-7 and A549 cells by Nic-Chi Np's was chiefly due to apoptosis cascade.

### 3.6. Cell morphology analysis by field-emission scanning electron microscopy

**Figure 6.**
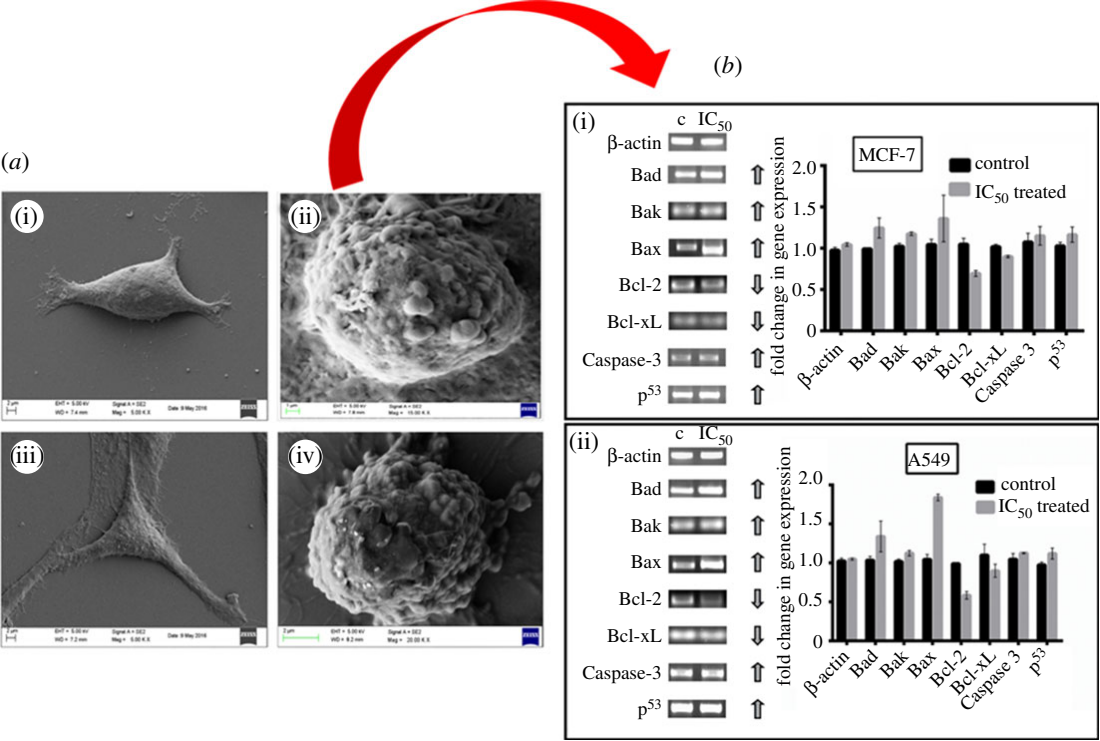
(*a*) Representative SEM images of untreated and Nic-Chi Np's treated cells of (i) control A549 cells; (ii) treated at 2 × IC_50_; (iii) control MCF-7 cells; (iv) treated at 2 × IC_50_; scale bar, 2 µm (untreated) and 1 µm (treated). (*b*) Semi-quantitative RT-PCR analysis of apoptotic signalling genes and their representative images. Lanes 1 and 2: untreated (c = control) and 7.5 µM IC_50_ treated (i) MCF-7 cells and (ii) A549 cells, with fold difference in gene expression. Results are represented as mean ± s.e. of the mean. *Significant difference from control (*p* < 0.05).

Nic-Chi Np's were added to MCF-7 and A549 in order to confirm their *in vitro* anti-cancerous efficacy. We found that in control cells both MCF-7 and A549 cells were healthy and well adhered to surface exhibiting normal morphology (figure 6*a*(i,iii)), but when they were treated with their respective 2 × IC_50_ (figure 6*a*(ii,iv)), they exhibited typical apoptotic morphological changes including membrane blebbing, chromatin condensation with margination of chromatin to the nuclear membrane, karyorrhexis (nuclear fragmentation) and formation of apoptotic bodies [29]. They became rounded, loosely attached to surface and showing the formation of apoptotic bodies. Figure 6*a* clearly depicts that the cell death was owing to apoptosis induced by Nic-Chi Np's not due to necrosis and revealed a potential anti-cancerous effect. The presence of chlorine (present in niclosamide) atoms was determined through elemental analysis of treated MCF-7 and A549 cells which further confirms that cell death in treated cells is due to apoptosis not necrosis (S4).

### 3.7. Gene expression studies by semi-quantitative RT-PCR analysis: niclosamide loaded chitosan nanoparticles trigger apoptotic gene signalling cascade

**Figure 7.**
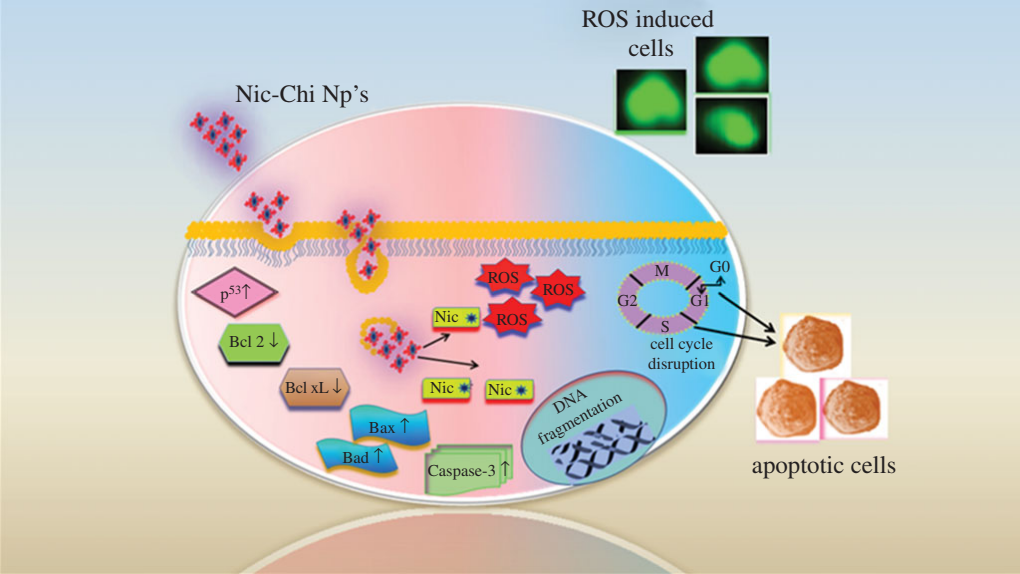
Schematic of nanoparticle cellular uptake and progressive apoptotic events involved in Nic-Chi Np's.

The anti-tumour efficacy of Nic-Chi Np's in breast and lung cancer cell lines was further assessed for apoptotic induction by semi-quantitative RT-PCR. Apoptosis occurs through either one of the two major pathways described as the intrinsic mitochondrial or extrinsic death receptor pathway [30]. Various signalling genes involved in the intrinsic and extrinsic pathways of apoptosis are shown in figure 7. The Nic-Chi Np-treated cells of both lung and breast cancer cell lines were showing apt amplification for apoptotic signalling genes in a semi-quantitative RT-PCR analysis. The pro-apoptotic signalling genes, including Bak (Bcl-2 homologous antagonist killer), Bax (Bcl-2 associated X), Bad (Bcl-2-associated death promoter), p^53^ (tumour suppressor protein p^53^), and Caspase-3, were amplified using gene specific primers as mentioned in §2. On the contrary, the anti-apoptotic genes were also amplified comprising proteins like Bcl-2 and Bcl-xL. The outcomes found to be that pro-apoptotic genes are upregulated while anti-apoptotic genes are downregulated indicate the apoptotic induction clearly. In order to reduce artefacts, we used β-actin as an internal control.

The functionality of niclosamide on mitochondrial fragmentation was exploited for anti-cancer studies. The mitochondrial fragmentation later induces the intrinsic pathways of apoptosis mediated by mitochondrial cytochrome *c*. The cytochrome *c* from the mitochondria is released into the cytosol, which leads to internal signalling cascade events. Bcl-2 family protein members are functioning as regulators of apoptosis. These comprise three subfamilies of protein, the anti-apoptotic subfamily (e.g. Bcl-xL, Bcl-2), the multi-domain pro-apoptotic subfamily (e.g. Bax, Bak) and the pro-apoptotic BH3-only subfamily (e.g. Bim, Bad) [31]. The pro-apoptotic proteins in the Bcl-2 family, including Bax and Bak, function on the outer mitochondrial membrane for promoting permeabilization and release of cytochrome *c*. These pro-apoptotic proteins are inhibited by the effective anti-functional properties of Bcl-2 and its relative Bcl-xL. These trans-membrane mitochondrial proteins [32] inhibit the flow of cytochrome *c* from inner mitochondria to the cytosol. In our study, the upregulation of Bax and Bad dictates the release of cytochrome *c* to the cytosol, whereas downregulation of Bcl-2 and Bcl-xL proteins evidently suggests the inhibitory effect of these proteins during cytochrome *c* flow, getting reduced through the mitochondrial voltage-dependent anion channel (VDAC). Furthermore, the upregulated Bak protein induces the interaction with the mitochondrial membrane and accelerates the opening of mitochondrial VDAC, leading to membrane potential loss and the efflux of cytochrome *c* to the cytosol [33].

P^53^ and Caspase-3 are common in the cellular growth and cell regulatory mechanisms. Both are highly expressed during the initial events of apoptosis. p^53^ initiates the apoptosis in cancer cells, and hence maintains homeostasis. The upregulated levels of p^53^ confirm apoptotic events were taking place while treating the A549 and MCF-7 cells with Nic-Chi Np's. Caspase-3, on the other hand, is an important protein to be expressed for the photolytic machinery during early occasions of apoptosis. Activated Caspase-3 will eventually initiate pro-caspase into active caspase transition. Higher levels of expressions of Caspase-3 as compared with untreated cells suggest that apoptosis was taking place during the treatment.

Considering the results of the semi-quantitative RT-PCR, it appeared that the Nic-Chi Np's have an apoptotic inducing potential which leads to cell death and high anti-cancer efficacy (figure 6*b*).

### 3.8. Niclosamide loaded chitosan nanoparticles induce oxidative stress by evoking intracellular reactive oxygen species

Chemiluminescent probes (luminol and lucigenin) and cell permeant fluorescent probe (DCFDDA) methods have been suggested by scientists to measure the free radical production in cells. Here, we monitored the redox state of the MCF-7 and A549 cells upon incubation with Nic-Chi Np's at their IC_50_, by detecting the increase in fluorescence. DCFH-DA is a cell permeant, the nonfluorescent precursor of DCF, acting as an intracellular probe for oxidative stress. Upon cellular diffusion, intracellular esterases cleave acetate group of DCFH-DA at the two ester bonds, producing a relatively polar and cell membrane-impermeable product, H_2_DCF. This nonfluorescent molecule accumulates intracellularly and subsequent oxidation yields the highly fluorescent product DCF [34,35].

**Figure 8.**
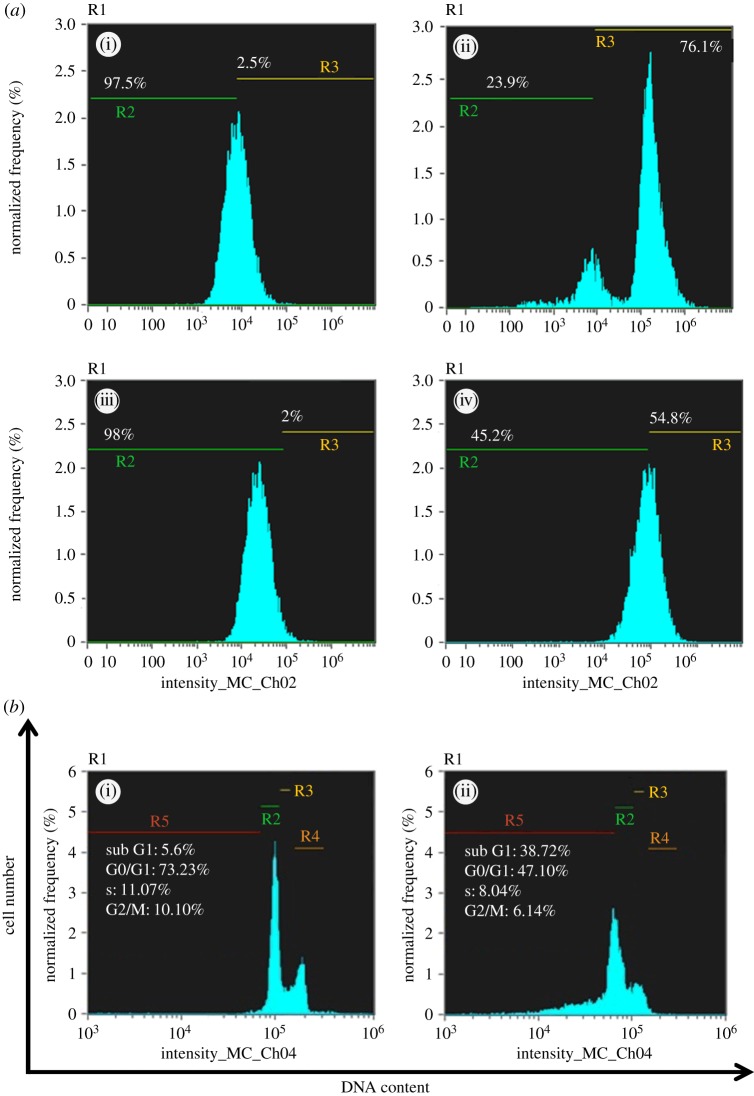
(*a*) Flow cytometric analysis of ROS production in MCF-7 and A549 cells: (i) MCF-7 control, i.e. untreated, (ii) treated with concentration at 2 × IC_50_; (iii) A549 control, (iv) treated with concentration at 2 × IC_50_. (*b*) Determination of cell-cycle distribution profile by flow cytometry analysis of PI-stained MCF-7 cells (i) untreated and (ii) treated at IC_50_ (7.5 µM).

ROS are chemically reactive species (O^2−^, OH·, H_2_O_2_) obtained as a natural by-product of aerobic metabolism. Dramatically elevated intracellular levels of ROS lead to significant damage to cell structures like lipids, proteins and DNA, this condition generally being referred to as oxidative stress. The induction of oxidative stress in MCF-7 and A549 cells initiates mitochondrial dysfunction and DNA fragmentation which eventually lead to apoptosis. In order to evaluate oxidative stress mediated apoptosis in MCF-7 and A549 cells, the extent of ROS generation was quantified at 2 × IC_50_ (17.5 µM for A549 and 15 µM for MCF-7) by DCFH-DA assay. The MCF-7 and A549 cell population fluorescence was gated on the basis of untreated control (figure 8*a*(i,iii)). From the flow cytometry data it is clearly shown that in control cells of MCF-7 (figure 8*a*(i)) 97.5% of cells are healthy whereas, when the cells are treated with their 2 × IC_50_, i.e. 15 µM, the apoptotic population increases drastically and only 23.9% (figure 8*a*(ii)) of total population remains healthy and around 76% undergoes apoptosis. Whereas, in A549 cells when they are treated with their 2 × IC_50_, i.e. 17.5 µM, the apoptotic population due to ROS induction became 54.8% (figure 8*a*(iv)). These results suggested that Nic-Chi Np's induced oxidative stress by evoking more ROS in MCF-7 than in A549.

Moreover, these results very much matched with the previous findings of MTT, wherein Nic-Chi Np's are readily taken up by MCF-7 cells and more susceptible than A549 cells. This result also supports our previous studies of Hoechst 33342 and Rho B staining and AO/EB dual staining where we demonstrated that at their IC_50_ values the MCF-7 and A549 cells were showing apoptosis.

Thus, taking into account the analysis of the H_2_DCFDDA assay for intracellular ROS, it can be concluded that the augmented cellular apoptosis was due to elevated oxidative stress.

### 3.9. Cell-cycle analysis of niclosamide loaded chitosan nanoparticle-treated cells

With our better understanding obtained from the above data of anti-cancer potential of Nic-Chi nano-carrier, there is now interest in cell-cycle analysis of MCF-7 cells as they seem to be more amenable for elucidating the apoptotic expression induced by Nic-Chi Np's.

Here, the data acquisition software (Amnis) records the fluorescence intensities (the integrated area of the electronic pulse signal) of 10^4^ cells per sample. The data are presented as cellular DNA content frequency histograms. PI is widely used in a univariate analysis of cellular DNA content by cell-cycle analysis method [36]. It stains whole cells or isolated nuclei via intercalation into the major groove of double-stranded DNA resulting in a highly fluorescent signal through excitation source (e.g. 488 nm argon ion laser) with a broad emission centred at 600 nm. As PI also stains double-stranded RNA, for optimal DNA resolution, RNase A was added to the staining solution in order to remove dsRNA [37].

Apoptotic cells often have high internucleosomal DNA fragment content due to the fact that low molecular weight fragmented DNA undergoes extraction during the cell staining procedure in aqueous solution. Some treated cells also lose DNA (chromatin) by shedding apoptotic bodies. Thus, within an apoptotic population, only a fraction of the DNA remains in cells. Apoptotic cells are then represented as cells with fractional (sub-G1) DNA content on the DNA content frequency histograms by the ‘sub-G1’ peak. From the analysis of DNA content frequency histograms, we estimated the proportions of cells (control and treated; figure 8*b*) in the sub-G1, G0/G1, S and G2M phases of the cell cycle. We observed exposure of Nic-Chi Np's at 7.5 µM in breast cancer cells results in disruption of cell-cycle phases and increases apoptotic cell population. As shown in figure 8*b*(ii), sub-G1 population increases approximately 7.5 times of control population, G0/G1 decreases from 73.23% to 47%, S from 11.07% to 8.04% and G2M from 10 to 6.14%. These results well support cytotoxicity data and ROS studies where cell death via apoptosis occurs. Our findings suggest that at very low concentration, anti-cancer drug niclosamide produces effective therapeutic efficacy, which may be due to high encapsulation efficiency of chitosan nano-carrier.

## 4. Conclusion

Water dispersible nanoformulations of niclosamide were prepared by glutaraldehyde cross-linking via covalent chemical interaction between positively charged amino groups of chitosan and aldehyde group of glutaraldehyde, which is confirmed by formation of Schiff base in FTIR analysis. Our study provides the first demonstration of the feasibility of using chitosan nanoparticles as cargo for hydrophobic niclosamide drug in anti-cancer therapy *in vitro*. The hydrophobic drug was easily loaded inside the nanocore and showed approximately 90% encapsulation. The positive zeta potential of chitosan nanoformulations facilitates them in crossing the plasma membrane barrier via ionic interaction with the negatively charged plasma membrane. *In vitro* cell studies further established that even at low concentration Nic-Chi Np's are able to induce ROS and apoptosis. From the semi-quantitative analysis of gene expression of A549 and MCF-7 cells, it is clear that there is an increase in expression of genes (p^53^, Caspase-3, Bax, Bad) and decreases in expression of Bcl family notably verified the association of Nic-Chi Np's in the cascade of apoptosis. Additionally, FE-SEM images illustrated the formation of apoptotic bodies and membrane blebbing upon incubation of Nic-Chi Np's in both cancer cell types. *In vitro* drug release studies clarify the potential of chitosan nanoparticles as an efficient cargo to release the drug cargo, i.e. niclosamide, under physiological conditions. Nic-Chi Np's due to their biodegradable and biocompatible nature, high therapeutic efficacy, and easy synthesis protocol can be easily exploited for *in vivo* and clinical studies. In near prospect, niclosamide and other water insoluble anti-cancer drugs can be easily delivered to the site of action by targeted chitosan nanoformulations and further allowing reduction of daily dose and cytotoxicity. Thus, Nic-Chi Np's may have a great potential even at low concentration for anti-cancer therapy. They can effectively improve the stability of niclosamide drug, which may replace or substitute more toxic anti-mitotic drugs in the near future.

## Data accessibility

The datasets supporting this article can be found at http://dx.doi.org/10.5061/dryad.k8j74 [38].
